# I MAY BE ALONE BUT I DON'T FEEL LONELY: INSIGHTS FROM THE ORAL NARRATIVES OF CHILDLESS CENTENARIANS

**DOI:** 10.1093/geroni/igz038.151

**Published:** 2019-11-08

**Authors:** Melinda Heinz, Alex J Bishop, Tanya Finchum

**Affiliations:** 1 Upper Iowa University, Fayette, Iowa, United States; 2 Oklahoma State University, Stillwater, Oklahoma, United States

## Abstract

Oral history narratives of nine childless centenarians (7 women, 2 men) from the Oklahoma 100 Year Life Project were reviewed to investigate loneliness. Oral history narratives were assessed qualitatively, using content analysis to determine themes. We predicted that childless centenarians would feel lonely due to “elder orphanhood.” Findings revealed little indication of loneliness. Centenarians admitted they voluntarily chose to remain childless due to raising siblings earlier in life or delaying marriage. However, most remained socially well-adjusted and connected to extended family, particularly nieces. When confronted by social network losses due to death or relocation, most adapted by actively seeking and forming new relationships. In some cases, childless centenarians remained gainfully employed and working. Childlessness does not appear to make centenarians lonely. Rather, purposeful pursuit of intrapersonal and interpersonal sources of lifelong emotional contentment may render childless centenarians immune from conditions of loneliness.

